# Supplementary material for: Relationship between Osteosarcopenia and Frailty in Patients with Chronic Liver Disease
Source: J Clin Med. 2020 Jul 26;9(8):2381. doi: 10.3390/jcm9082381 (PMC7465351; doi:10.3390/jcm9082381)
Supplement: Supplementary file 1 [file jcm-09-02381-s001.pdf]

Supplementary Materials:

Table S1. Characteristics of patients with and without sarcopenia/osteoporosis.

Variable	Osteoporosis (−) Sarcopenia (−)	Osteoporosis (+) Sarcopenia(−)	Osteoporosis (−) Sarcopenia (+)	Osteoporosis(+) Sarcopenia (+)	p-value
Patients, n (%)	168 (57.7)	46 (15.8)	28 (9.6)	49 (16.8)	
Male, n (%)	91 (54.2)	14 (30.4)	16 (57.1)	16 (32.7)	0.003
Age (years)	65.0 (54.0–71.8)	72.0 (64.5–79.0)	75.0 (63.8–76.8)	76.0 (72.5–81.0)	<0.001
BMI (kg/m^2)	24.4 (21.9–26.6)	22.8 (20.6–25.6)	22.1 (19.4–23.3)	20.4 (19.0–22.2)	<0.001
Liver cirrhosis, n (%)	81 (48.2)	20 (43.5)	19 (67.9)	31 (63.3)	0.055
Etiology					
HBV/HCV/Alcohol/PBC/other, n	27/37/37/32/35	9/20/4/7/6	0/14/6/6/2	5/21/5/14/4	0.002
Total bilirubin (mg/dL)	0.7 (0.5–1.0)	0.8 (0.5–1.0)	0.9 (0.5–1.6)	0.6 (0.4–0.8)	0.025
Albumin (g/dL)	4.1 (3.7–4.4)	4.1 (3.8–4.4)	3.8 (3.3–4.1)	4.0 (3.6–4.3)	0.022
Prothrombin time INR	1.05 (0.97–1.15)	1.03 (0.97–1.10)	1.13 (0.99–1.25)	1.06 (0.97–1.11)	0.186
M2BPGi (C.O.I)	1.32 (0.71–3.31)	1.59 (0.84–3.48)	3.49 (1.02–7.57)	1.99 (1.37–2.96)	0.003
IGF-1 (ng/mL)	76 (54–101)	59 (46–84)	49 (33–77)	49 (41–64)	<0.001
Zinc (μg/dL)	69 (60–78)	69 (61–80)	62 (48–73)	66 (56–76)	0.017
BCAA (μmol/L)	434 (385–488)	388 (356–456)	354 (310–426)	333 (293–401)	<0.001
TRACP-5b (mU/dL)	375 (293–497)	449 (346–651)	476 (418–629)	463 (311–596)	0.002
P1NP (ng/mL)	48 (34–65)	56(41–79)	62 (34–83)	46 (34–76)	0.206
PTH-intact (pg/mL)	44 (34–55)	49 (39–63)	38 (27–57)	51 (38–85)	0.011
SMI (kg/m2)	7.09 (6.34–7.79)	6.19 (5.79–6.78)	5.81 (5.28–6.48)	5.12 (4.74–5.52)	<0.001
Handgrip strength (kg)	28.3 (21.9–35.5)	19.7 (17.6–27.8)	18.3 (16.5–24.1)	15.1 (13.1–17.8)	<0.001
Lumbar spine BMD (g/cm^2)	1.15 (1.02–1.28)	0.87 (0.78–1.04)	1.11 (0.98–1.20)	0.85 (0.75–0.95)	<0.001
Femoral neck BMD (g/cm^2)	0.85 (0.77–0.94)	0.64 (0.58–0.68)	0.80 (0.73–0.87)	0.62 (0.56–0.65)	<0.001
Total hip BMD (g/cm^2)	0.91 (0.83–0.98)	0.69 (0.63–0.73)	0.87 (0.79–0.91)	0.66 (0.58–0.71)	<0.001
Frailty, n (%)	10 (6.0)	13 (28.3)	19 (67.9)	39 (79.6)	<0.001
Low gait speed (m/s), n (%)	23 (13.7)	15 (32.6)	18 (64.3)	39 (79.6)	<0.001
Vertebral fracture, n (%)	26 (15.5)	17 (37.0)	6 (21.4)	29 (59.2)	<0.001

Values are shown as median (interquartile range) or number (percentage). Statistical analysis was performed using the chi-squared test or the Kruskal-Wallis test, as appropriate. BCAA, branched-chain amino acids; BMD, bone mineral density; BMI, body mass index; HBV, hepatitis B virus; HCV, hepatitis C virus; IGF-1, insulin-like growth factor 1; INR, international normalized ratio; M2BPGi, Mac-2 binding protein glycosylation isomer; PBC, primary biliary cholangitis; P1NP, procollagen type N-terminal propeptide; PTH, parathyroid hormone; SMI, skeletal muscle mass index; TRACP-5b, tartrate-resistant acid phosphatase 5b.

Table S2. Univariate logistic analysis for predictors associated with osteosarcopenia.

Variable	OR (95% CI)	p-value
Male	0.485(0.254–0.927)	0.029
Age (years)	1.094(1.053–1.136)	<0.001
BMI (kg/m^2)	0.767(0.687–0.857)	<0.001
Liver cirrhosis	1.751(0.930–3.298)	0.083
Etiology	0.925(0.730–1.171)	0.925
Total bilirubin (mg/dL)	0.752(0.411–1.378)	0.357
Albumin (g/dL)	0.672(0.379–1.190)	0.173
Prothrombin time INR	0.371(0.040–3.444)	0.383
M2BPGi (C.O.I)	0.973(0.882–1.075)	0.595
IGF-1 (ng/mL)	0.974(0.961–0.987)	<0.001
Zinc (μg/dL)	0.993(0.973–1.014)	0.537
BCAA (μmol/L)	0.993(0.989–0.996)	<0.001
TRACP-5b (mU/dL)	1.001(1.000–1.002)	0.240

Total P1NP (ng/mL)	1.001(0.996–1.006)	0.690
PTH-intact (pg/mL)	1.019(1.009–1.029)	<0.001
Frailty	18.571(8.596–40.121)	<0.001
Vertebral fracture	5.711(2.981–10.943)	<0.001

BCAA, branched-chain amino acids; BMI, body mass index; CI, confidence interval; IGF-1, insulin-like growth factor 1; INR, international normalized ratio; M2BPGi, Mac-2 binding protein glycosylation isomer; OR, odds ratio; P1NP, procollagen type N-terminal propeptide; PTH, parathyroid hormone; TRACP-5b, tartrate-resistant acid phosphatase 5b.

Table S3. Characteristics of patients with and without pre-frailty/frailty.

Variable	Non-frailty	Pre-frailty	Frailty	p-value
Patients, n (%)	98 (33.7)	112 (38.5)	81 (27.8)	
Male, n (%)	48 (49.0)	58 (51.8)	31 (38.3)	0.160
Age (years)	66.5 (56.0–72.0)	67.5 (56.3–73.0)	76.0 (69.5–80.0)	<0.001
BMI (kg/m^2)	24.4 (21.7–26.9)	23.5 (21.1–25.7)	21.5 (19.8–24.5)	<0.001
Liver cirrhosis, n (%)	36 (36.7)	59 (52.7)	56 (69.1)	<0.001
Etiology				
HBV/HCV/Alcohol/PBC/other, n	22/26/15/16/19	12/30/28/27/15	7/36/9/16/13	0.006
Total bilirubin (mg/dL)	0.8 (0.6–1.0)	0.7 (0.5–1.0)	0.7 (0.5–1.0)	0.242
Albumin (g/dL)	4.2 (3.9–4.4)	4.0 (3.6–4.3)	3.9 (3.4–4.3)	<0.001
Prothrombin time INR	1.03 (0.97–1.15)	1.06 (0.97–1.10)	1.07 (0.99–1.17)	0.256
M2BPGi (C.O.I)	1.25 (0.69–1.13)	1.06 (0.97–1.16)	2.33 (1.29–4.70)	<0.001
IGF-1 (ng/mL)	79 (58–107)	63 (47–89)	54 (41–69)	<0.001
Zinc (μg/dL)	71 (64–79)	68 (59–78)	63 (51–75)	0.002
BCAA (μmol/L)	436 (379–493)	410 (363–468)	370 (308–412)	<0.001
TRACP-5b (mU/dL)	404 (307–502)	393 (317–569)	458 (323–592)	0.160
P1NP (ng/mL)	48 (34–60)	51 (36–78)	49 (33–79)	0.207
PTH-intact (pg/mL)	44 (35–57)	48 (35–57)	48 (33–68)	0.662
SMI (kg/m2)	7.02 (6.21–7.80)	6.71 (5.95–7.42)	5.65 (4.97–6.24)	<0.001
Handgrip strength (kg)	28.5 (22.3–35.3)	24.2 (19.5–31.4)	16.4 (13.2–20.2)	<0.001
Lumbar spine BMD (g/cm^2)	1.12 (0.96–1.26)	1.09 (0.94–1.24)	0.93 (0.82–1.11)	<0.001
Femoral neck BMD (g/cm^2)	0.83 (0.71–0.91)	0.80 (0.71–0.92)	0.65 (0.60–0.72)	<0.001
Total hip BMD (g/cm^2)	0.87 (0.77–0.97)	0.85 (0.76–0.96)	0.70 (0.63–0.80)	<0.001
Low gait speed (m/s), n (%)	0 (0.0)	20 (17.9)	75 (92.6)	<0.001
Osteosarcopenia, n (%)	1 (1.0)	9 (8.0)	39 (48.1)	<0.001
Vertebral fracture, n (%)	16 (16.3)	22 (19.6)	40 (49.4)	<0.001

Values are shown as median (interquartile range) or number (percentage). Statistical analysis was performed using the chi-squared test or the Kruskal-Wallis test, as appropriate. BCAA, branched-chain amino acids; BMD, bone mineral density; BMI, body mass index; HBV, hepatitis B virus; HCV, hepatitis C virus; IGF-1, insulin-like growth factor 1; INR, international normalized ratio; M2BPGi, Mac-2 binding protein glycosylation isomer; PBC, primary biliary cholangitis; P1NP, procollagen type N-terminal propeptide; PTH, parathyroid hormone; SMI, skeletal muscle mass index; TRACP-5b, tartrate-resistant acid phosphatase 5b.

Table S4. Univariate logistic analysis for predictors associated with frailty.

Variable	OR (95% CI)	p-value
Male	0.608 (0.360–1.027)	0.063
Age (years)	1.089 (1.056–1.123)	<0.001
BMI (kg/m^2)	0.875 (0.811–0.943)	0.001
Liver cirrhosis	2.712 (1.574–4.672)	<0.001
Etiology	0.979 (0.805–1.190)	0.830

Total bilirubin (mg/dL)	1.077 (0.733–1.583)	0.704
Albumin (g/dL)	0.375 (0.227–0.621)	<0.001
Prothrombin time INR	2.181 (6.397–11.970)	0.369
M2BPGi (C.O.I)	1.124 (1.041–1.214)	0.003
IGF-1 (ng/mL)	0.980 (0.970–0.989)	<0.001
Zinc (μg/dL)	0.974 (0.956–0.992)	0.006
BCAA (μmol/L)	0.991 (0.988–0.995)	<0.001
TRACP-5b (mU/dL)	1.001 (1.000–1.002)	0.077
Total P1NP (ng/mL)	1.002 (0.997–1.006)	0.422
PTH-intact (pg/mL)	1.009 (1.001–1.018)	0.032
SMI (kg/m2)	0.266 (0.186–0.381)	<0.001
Handgrip strength (kg)	0.781 (0.731–0.834)	<0.001
Lumbar spine BMD (g/cm^2)	0.042 (0.011–0.162)	<0.001
Femoral neck BMD (g/cm^2)	0.000 (0.000–0.004)	<0.001
Total hip BMD (g/cm^2)	0.001 (0.000–0.005)	<0.001
Osteosarcopenia	18.571 (8.596–40.121)	<0.001
Vertebral fracture	4.416 (2.523–7.728)	<0.001

BCAA, branched-chain amino acids; BMD, bone mineral density; BMI, body mass index; CI, confidence interval; IGF-1, insulin-like growth factor 1; INR, international normalized ratio; M2BPGi, Mac-2 binding protein glycosylation isomer; OR, odds ratio; P1NP, procollagen type N-terminal propeptide; PTH, parathyroid hormone; SMI, skeletal muscle mass index; TRACP-5b, tartrate-resistant acid phosphatase 5b.
